# Correlation of Six-Minute Walking Performance with Quality of Life is Domain- and Gender-Specific in Healthy Older Adults

**DOI:** 10.1371/journal.pone.0117359

**Published:** 2015-02-19

**Authors:** Andrey Jorge Serra, Paulo de Tarso Camillo de Carvalho, Fernanda Lanza, Camila de Amorim Flandes, Shirley Cardoso Silva, Frank Shiguemitsu Suzuki, Danilo Sales Bocalini, Erinaldo Andrade, Cezar Casarin, José Antonio Silva

**Affiliations:** 1 Post-Graduation at Biophotonic Applied to Health Science, Nove de Julho University (UNINOVE), São Paulo, SP, Brazil; 2 Post-graduation Program in Rehabilitation Sciences, Nove de Julho University (UNINOVE), São Paulo, SP, Brazil; 3 Post-Graduation at Physical Education, São Judas Tadeu University (USJT), São Paulo, SP, Brazil; 4 Post-Graduation at Medicine, Nove de Julho University (UNINOVE), São Paulo, SP, Brazil; University of Akron, UNITED STATES

## Abstract

We analyzed the relationship between performance on the 6-min walk test (6MWT) and health-related quality of life (HRQoL) in older subjects. Our secondary aim was to determine the distance to be completed on the 6MWT for the subject to achieve a score of 50 on the Short Form (36) Health Survey (SF-36). Associations were tested using linear correlation and multivariate linear regression. Participants were 130 healthy older individuals. The predictive performance of the 6MWT based on an SF-36 score of 50 was assessed using a receiver operating characteristic curve and its area under curve (AUC). Associations were observed between physical functioning, role-emotional, social functioning, vitality, general health score, and 6MWT performance in women, after adjusting for confounding variables (coefficients: 0.57, 0.38, 0.40, and 0.46, respectively; p < 0.05). No association was found for men. The distance for the 6MWT to predict an SF-36 score of 50 was 481 m for men in the physical functioning (AUC: 0.79) and role-physical (AUC: 0.84) domains, and 420 m for women in role-emotional (AUC: 0.75), role-physical (AUC: 0.80), and general health (AUC: 0.80) domains. Our results indicate that superior 6MWT performance may be associated with better HRQoL in several domains in only healthy older women. No association between 6MWT performance and role-emotional, mental health, or vitality domains was found. We suggest that a score of 50 is represented by a 6MWT distance of 481 m for men and 420 m for women, at least in the role-physical domain.

## Introduction

The older population is increasing rapidly around the world. Epidemiological estimates indicate that two billion people will be over 60 years old in 2050 [1]. This is an important issue because the aging process reduces functional fitness and increases individuals’ risk of contracting or developing many diseases [2,3].

In light of these data, means of mitigating functional deficits and improving health-related quality of life (HRQoL) in the elderly are required. Physical activity can play a key role in this process. An active lifestyle has been shown to prevent cardiovascular diseases, maintain independence, and improve functional capacity in older people [4,5]. Moreover, evidence has shown that increased PA is associated with improvement in HRQoL.

The six-minute walk test (6MWT) has been widely used to assess functional capacity due to the simplicity of its implementation, ease of interpretation, and representativeness of activity in daily life. The 6MWT performance has become the most common method for evaluating functional capacity in individuals with a variety of health conditions [6,7]. Hsieh et al. reported a significant correlation between distance walked in the 6MWT and HRQoL in post-donor liver transplantation [8]. A similar finding was reported in a study of patients with pulmonary emphysema [9]. These findings indicate a relationship between physical activity and HRQoL.

To our knowledge, no previous studies have analyzed a direct relationship between functional capacity and HRQoL in older individuals without a reported disease. We hypothesized that a higher 6MWT performance (in terms of distance walked) is associated with a better HRQoL in older individuals without chronic diseases or cognitive impairment. We also determined the minimum distance on the 6MWT that would reliably predict a score of 50 on the Short Form (36) Health Survey (SF-36), which indicates good quality of life [10,11].

## Materials and Methods

### Study setting

The experimental protocol followed the principles expressed in the Declaration of Helsinki. The Uninove Research Ethics Committee granted ethical approval (Process: 458917), and all participants provided written informed consent. A total of 235 sedentary participants were recruited from a center that promotes physical activity for health benefits [12]. Inclusion criteria were as follows: (a) >60 years old, (b) free of any chronic disease or cognitive impairment, (d) no regular alcohol intake, (e) non-smoker, (f) functionally independent regarding daily physical activity, (g) a resident of the targeted area of the city, (h) no marked obesity or physical disability, (i) no report of bodily pain, and (j) no registered blindness or severe hearing impairment. The final sample comprised 60 men (aged 70 ± 7 years) and 70 women (aged 69 ± 7 years). Assessment was conducted in an indoor area such as a multipurpose room or gymnasium. Participants were assessed separately and were instructed to wear appropriate clothing and footwear, to eat a light meal approximately 2 hours before testing, to avoid drinking alcoholic beverages in the preceding 24 hours, and to not perform PA during the 24 hours prior to assessment.

### Anthropometric analysis

Body composition variables were assessed as previously described with a Cardiomed stadiometer (WCS model, PR, Brazil) and a balance scale (Personal Line 150, Brazil) [13].

### General self-reported HRQoL

A validated Brazilian version of the SF-36 was used to evaluate HRQoL [14]. The SF-36 assesses several domains of health-related HRQoL, including physical functioning and general health perceptions. The reliability of the SF-36 for older subjects has been determined to be .92 for physical functioning and .78 for general health [15]. Seven scale domains were analyzed: physical functioning, role-emotional, role-physical, social functioning, mental health, vitality, and general health. Each scale was linearly transformed into a 0–100 scale, with higher scores representing better health status and functioning. In keeping with previous research, the value of 50 was considered to indicated good quality of life [10,11], and this value was used as a cutoff point.

### 6MWT

The 6MWT was administered by a well-trained physical appraiser blinded for HRQoL results. Participants were instructed to walk from one end of a 20-meter course to the other and back again for 6 min, according to ATS/ERS guidelines [6]. Participants were encouraged in a standardized manner to walk as far as possible at a reasonable amount of effort. Total distance walked was recorded as the participant’s predicted value [16]. Participants’ predicted value was used to establish a baseline of functional capacity for the studied population. Pain or discomfort in upper and/or lower limbs, syncope, chest pain, and dyspnea were recorded continuously during the evaluation. Heart rate was recorded during the test; and maximal heart rate was derived from the formula: maximal heart rate = 220 – age in years.

### Statistical Analysis

Analyses were performed using SPSS 14.0. The Shapiro–Wilk test was used to verify data distributions. Variable comparisons were carried out using an unpaired Student’s *t*-test. Proportions were analyzed using the chi-square test. Pearson’s correlation coefficient was calculated to evaluate the relationship between HRQoL and 6MWT performance, and multivariate linear regression models were used to assess the independent relationships between HRQoL and 6MWT. Receiver operating characteristic (ROC) curve analysis was used to determine whether distance walked on the 6MWT could predict SF-36 scores. The area under the curve (AUC) was used to analyze prediction accuracy. An AUC of 1 indicates that the test perfectly differentiates scores above and below the cut-off point, and an AUC of less than 0.7 means that the test does so poorly. Data were expressed as mean ± SD, and the significance threshold was ≤ 0.05.

## Results

Body mass index was similar between genders (men: 25 ± 3, women: 25 ± 3 kg/m^2^, p = 0.3). Most subjects performed satisfactorily on the 6MWT; predicted distance was achieved for 95 ± 20% of participants. Men walked a greater distance (484 ± 125 m) and a higher proportion achieved the predicted distance (99 ± 20%) compared to women (412 ± 89 m; 91 ± 18%) (p < 0.01). There were no significant differences between genders for HRQoL domains (data not shown).

Significant correlations were found between all HRQoL domains and 6MWT performance in the overall sample (Table 1). For men, the domains correlated with 6MWT performance were physical functioning, role-physical, and social functioning. In women, 6MWT performance was significantly correlated with all domains of HRQoL except mental health.

**Table 1:** Association between 6-min walk test performance and quality of life domains.

**Domain**	**Linear correlation**						**Multivariable Linear regression**					
	**Overall (N = 130)**		**Men (N = 60)**		**Women (N = 70)**		Overall (N = 130)*		**Men (N = 60)^#^**		**Women (N = 70)^#^**	
	**r**	**P value**	**r**	**P value**	**r**	**P value**	**β**	**P value**	**β**	**P value**	**β**	**P value**
PF	0.38	<0.001	0.33	0.004	0.57	<0.001	0.25	0.02	0.29	0.2	0.57	<0.001
RE	0.16	0.02	0.10	0.2	0.35	0.001	-0.002	0.9	0.31	0.8	0.19	0.2
RP	0.33	<0.001	0.60	<0.001	0.27	0.01	0.28	0.01	0.29	0.08	0.38	0.02
SF	0.27	0.001	0.27	0.01	0.37	0.001	0.10	0.3	0.06	0.7	0.40	0.01
MH	0.16	0.02	0.17	0.09	0.06	0.2	0.02	0.8	0.32	0.7	0.004	0.9
VT	0.44	<0.001	0.20	0.054	0.33	0.002	0.21	0.06	0.34	0.053	0.23	0.1
GH	0.18	0.01	-0.20	0.06	0.38	0.006	0.06	0.5	0.04	0.8	0.46	0.01

PF, physical functioning; RE, role-emotional; RP, role-physical; SF, social functioning; MH, mental health; VT, vitality; GH, general health.

*Adjusted for sex, body weight, height, and predicted distance.

^*#*^Adjusted for body weight, height and predicted distance.

Adjusted linear regression models of the relationship between 6MWT performance and HRQoL showed a significant association only for the physical functioning and role-physical domains when potential confounding variables (gender, body weight, height) were included on overall sample (Table 1). Thus, the inclusion of potential confounding variables in the model changed the results of linear correlation tests for several domains. This issue was more pronounced in men, in whom standardized regression coefficients (β) were not significant. In women, adjusted models changed the results of linear correlation tests for the role-emotional and vitality domains.

To perform ROC curve analysis, a score of 50 on the SF-36 was used as a cutoff (Table 2). Test sensitivity and specificity were set at the median 6MWT performance, measured in meters walked. First, the overall sample was tested, and sensitivity and specificity was set at the median distance walked, which was 447 m. Only for the general health and role-physical domains was the AUC over 0.70, despite low sensitivity. Specifically for general health domain, when the cutoff of 447 m in the 6MWT was used, 64% of all participants who scored over 50 on the SF-36 were correctly identified, and 30% of participants who walked 447 m did not score above 50 on the SF-36. For men, median distance walked was 481 m, and AUC was over 0.70 for the physical functioning and role-physical domains. For women, median distance walked was 420 m. The AUC was calculated for the role-emotional, role-physical, and general health domains.

**Table 2:** Accuracy of 6MWT distance in predicting health-related quality of life scores.

	Median TC6 (m)	Sensitivity	Specificity	AUC (95%CI)	P value
Overall sample	447				
PF		0.58	0.26	0.67 (0.57–0.76)	0.004
RE		0.47	0.57	0.36 (0.26–0.46)	0.08
RP		0.56	0	0.81 (0.72–0.90)	<0.0001
SF		0.50	0.50	0.55 (0.42–20.69)	0.5
MH		0.56	0.35	0.61 (0.52–0.70)	0.04
VT		0.51	0.41	0.56 (0.46–0.66)	0.2
GH		0.64	0.30	0.71 (0.62–0.80)	<0.001
Men	481				
PF		0.62	0.13	0.79 (0.67–0.90)	<0.001
RE		0.21	0.58	0.27	0.01
RP		0.54	0	0.84 (0.72–0.95)	0.01
SF		0.53	0.28	0.62 (0.48–0.76)	0.2
MH		0.56	0.31	0.67 (0.54–0.80)	0.03
VT		0.56	0.31	0.67 (0.54–0.80)	0.03
GH*		---	---	---	---
Women	420				
PF		0.54	0.29	0.56 (0.42–0.71)	0.4
RE		0.55	0.25	0.75 (0.53–0.97)	0.02
RP		0.54	0.11	0.80 (0.67–0.93)	0.004
SF		0.49	0.33	0.62 (0.33–0.92)	0.4
MH		0.52	0.41	0.54 (0.41–0.68)	0.5
VT		0.49	0.46	0.49 (0.34–0.65)	0.9
GH		0.85	0.39	0.75 (0.61–0.88)	0.004

An SF-36 score of 50 was used, and the sensitivity and specificity of the test were set at the median 6MWT walk distance.

*There were no scores lower than 50 for quality of life on the general health domain; therefore, ROC curve analyses were not carried out. IC, confidence interval; PF, physical functioning; RE, role-emotional; RP, role-physical; SF, social functioning; MH, mental health; VT, vitality; GH, general health.

## Discussion

The main finding of this study was that 6MWT performance may be associated with HRQoL in a selected cohort of older participants. The association between 6MWT performance and HRQoL was also found to be gender-specific; higher 6MWT performance was associated with higher scores for the physical functioning, role-physical, social functioning, and general health domains in women.

Our findings agree with previous studies that have reported an association between functional fitness and self-reported HRQoL in older participants; that is, a superior 6MWT performance was associated with better physical functioning [17,18]. We extend these findings to show that a better 6MWT performance may also be associated with increased scores in the role-physical, social functioning, and general health domains.

It has been shown that body mass index, educational level, and comorbidities are associated with HRQoL [19,20]. Therefore, we selected a suitable sample and included covariates (e.g., sex, body weight, and height) in the regression models to explore independent associations. Notably, we observed significant associations among women but not men. Moreover, even among women, certain domains (role-emotional, mental health and vitality) were not significantly associated with 6MWT performance. It is not clear how to interpret these findings; this interpretational difficulty points to the issue implicit in the fact that there is currently no single theoretical approach capable of clarifying the underlying mechanisms of all the domains of HRQoL (e.g., family life, socioeconomic status, lifestyles, living arrangements, loneliness, race, and widowhood) [21–22]. The possibility that these variables influenced our analysis cannot be excluded.

Finally, further studies are required to determine a target predictive distance for a SF-36 score of 50. Our AUC data did not identify a perfectly accurate cut-off (AUC = 1) for both genders. This is important because a cut-off score of 50 has proved useful when interpreting differences between scales in the SF-36 survey [23–25]. It is therefore possible to use predictive 6MWT scores to analyze individual domains of HRQL.

## Conclusion

Our findings suggest that a better 6MWT performance may be associated with improved self-reports of several HRQoL domains in older women. However, further studies should further examine the minimum walking distance that will predict scores above 50 on the SF-36.

### Perspective and limitations

This study has an important implication—aerobic physical fitness can be directly associated with HRQoL in healthy older women. The results obtained did not suggest the same relationship among men. The reasons for these findings are not clear. Further research evaluating hypotheses that would explain these findings is required. Future research should aim to determine whether scores on each SF-36 domain are sensitive to gender, since this sensitivity could influence the association between HRQoL and aerobic fitness in men. Moreover, it has been shown that a high value for maximal aerobic power (VO_2_max) is associated with better cardiovascular health in older individuals [26]. Thus, the association between VO_2_max and specific HRQoL domains should be analyzed. The current study is also unable to determine whether the observed associations can be extended to among individuals residing outside the data collection site; hence, future investigations should aim to determine whether an association between HRQoL and aerobic physical fitness holds among older women demographic status and lifestyle is different to those who participated in this study.

Some limitations must be considered. Exclusion criteria were very strict; this limits the generalisability of the obtained results. This strict selection process was intended to control for several biases that may otherwise have limited the validity of the analysis. Another limitation of the study is that socioeconomic status of participating women was not controlled. Williams et al. [27] have previously shown that women with higher socioeconomic status report better health than those with lower status. Although the female participants resided in a single area of the city, it was not possible to determine their socioeconomic status. Finally, certain limitations are inherent in the SF-36 survey. Symptoms and problems linked to specific conditions (e.g., problems with sleep, cognitive functioning, sexual functioning, social functioning, self-esteem, eating, and the disruption of recreation/hobbies, as well as health-related distress) are not analyzed by the SF-36, because the survey is intended as a generic measure [11]. Moreover, the SF-36 may be limited to assessments of elderly subjects with higher cognitive and physical functioning [28].

The possibility that the above factors influenced the assessment of HRQoL cannot be excluded. Our results are likely to be broadly transferable to populations evaluated with the same measurement tool used in the present study.
